# Camel biodiversity—and how to conserve it

**DOI:** 10.1093/af/vfac042

**Published:** 2022-08-12

**Authors:** Ilse Köhler-Rollefson

**Affiliations:** Policy Department, League for Pastoral Peoples, Ober-Ramstadt 64372, Germany

**Keywords:** adaptation, biodiversity, camel, food security, genetic erosion, pastoralism

**Implications**

- The existing genetic diversity camels is the result of traditional breeding practices and culturally embedded utilization patterns of camel breeding ethnic groups.
- Many camel breeding communities experience threats to their traditional management systems due to alienation of customary grazing areas and unfavorable policy environments.
- “Modern” breeding practices such as embryo transfer and cloning narrowing the camel gene pool.
- To maintain genetic diversity in camels and their ability to produce food in challenging environments, in-situ conservation by camel herding communities needs strengthening.
- The affluent countries eroding camel biodiversity should support communities in poor countries that conserve camel biodiversity.

## Introduction

DAD-IS, the Domestic Animal Diversity Information System maintained at the FAO, documents 89 breeds of one-humped camels and 14 breeds of Bactrian camels. Of the dromedary breeds, 47 are at home in Africa, 14 in Asia, and 23 in the Middle East. Only 2 breeds are classified as transboundary. Nine of the Bactrian breeds are in Asia, 3 in Europe, and 2 are transboundary (DAD_IS, 2022).

## Origin of Camel Biodiversity and History of Camel Breeding

These existing camel breeds are the result of “traditional,” “indigenous” breeding practices of camel breeding communities or ethnic groups that comprise a number of mechanisms by which separate gene pools are established and manipulated according to cultural preferences. Camel pastoralists differ in how they use camels. Among the Cushitic groups in the Horn of Africa and East Africa, the emphasis is on using camels for milk which formed the staple for groups such as the Somali. Among the Bedouin of the Arabian Peninsula, camels were used pragmatically for milk, meat, and transportation. In Asia, camels were used mostly for transportation, although for some groups use of milk was and is also important.

Camel breeds are often named after the ethnic group they are associated with, for instance in Kenya, there are the Somali, Gabra, Rendille, and Turkana breeds, in Sudan, there are the Bishareen, the Rashaida, and the Kababish breeds. However, in India, scientific classification bases breed names on the geographical area or erstwhile kingdom they can be found in.

**Figure 1.**
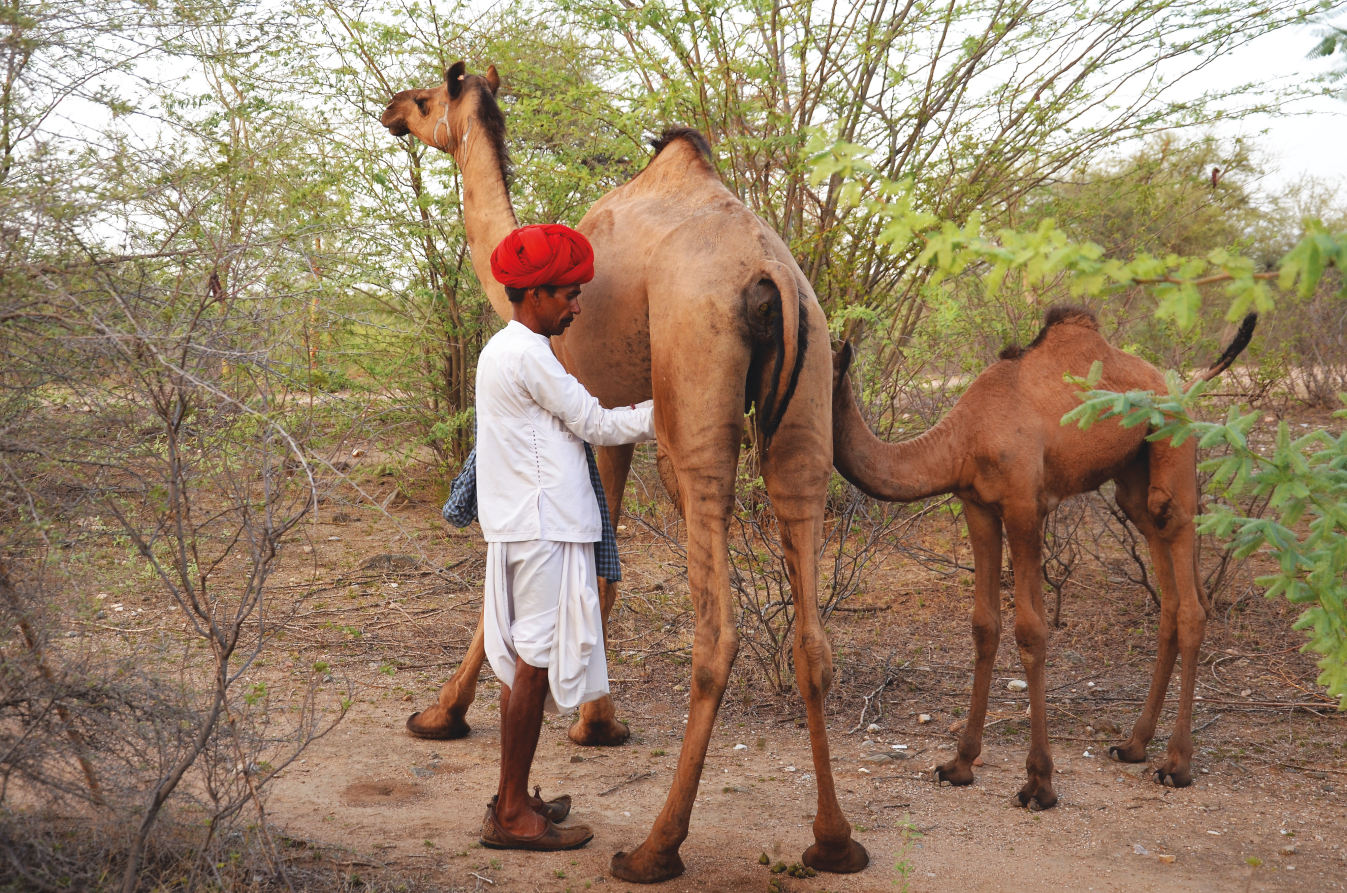
Camel pastoralists, such as the Raika of Rajasthan in India, have very concrete selection criteria that differ between ethnic groups and may change in response to markets and external conditions. While there was a market for draught camels, the Raika was selected for body size and physical strength, but now gives more weight to milk yields and milkability, as a market for camel dairy products develops.

A general rule in camel pastoralist societies was that female camels could not be sold to anybody outside the community; they were passed on within the community, from generation to generation or exchanged as dowry and bridewealth. This is an important factor in breed development processes. While camel pastoralists did not keep written records or registered animals in herd books, they memorized the ancestry of their camels and often were sticklers for pure breeding. Each community also had its individual concepts of beauty and desirable physical characteristics, selecting for different colors, and body formations (Köhler-Rollefson, 1993) (Figure 1).

At the same time, camel biodiversity is not only a question of genes but also of management. The capacity to thrive in specific eco-zones is also a result of training and acclimatization. The Amar’ar Beja in Sudan differentiate between *shallagea* camels used as pack animals, *aiiyit* suitable for riding, and *matiat* for racing. The three types develop through adaptation of young camels to specific pastures with nutritionally different grazing rather than selective breeding. The riding and racing types turn into shallagea types if they are raised on lush pasture at the coast (Hjort and Dahl, 1984).

The Somali and Rendille of Northern Kenya manage their camels differently, resulting in quite distinct types of camels. The Somalis try to create ideal conditions for their camels, providing them with optimal access to pasture and ensuring frequent watering opportunities. The Rendille seek to harden their camels by systematically restricting their water intake from an early age and allowing them less time to graze. As a result, the Somali camels are large, heavy, and good milk producers whereas the Rendille breed is small and extremely tough (Schlee, 1988).

Pastoralists in India report that it is difficult to transfer camels from one type of environment to another, as young camels learn their foraging behavior from their mothers. If moved from the Thar Desert to the Aravalli Hills, they literally do not know which plants to eat and lose condition, often taking years to acclimatize.

Until very recently, camels were never subjected to the kind of breeding that is conventionally deemed “scientific,” entailing metrical documentation of physical parameters and performance recording. Yet, the diversity created by means of indigenous knowledge is impressive, ranging from slender long-legged breeds suitable for racing to slow and heavy dairy animals as well as very tall and strong beasts suitable for demanding physical work as draught or baggage animals.

Unfortunately, there are currently two distinct processes at work that render this diversity very much under threat. These began during the 20th century and have developed further momentum during the 21st century.

For one, many of the camel breeding pastoralist communities are under threat. This is especially the case in India where the Raika community has lost customary grazing rights to its monsoon season pastures in the Aravalli Hills, in addition to facing alienation of other grazing areas through fencing, irrigation, use for green energy, and military purposes. In combination with legal restrictions on the export and use for meat, this scenario has rendered camel breeding economically unprofitable leading to a massive decline in the camel population of Rajasthan, the state that is home to about 80% of India’s camels (LPPS, 2010). The situation is slightly better in Gujarat where camels are well integrated with crop cultivation and the policy environment is less prohibitive.

East Africa is the region with the largest camel population, and here the sector seems to be thriving, as reflected in the substantial export of camels for meat to Arabian countries, as well as a dynamic camel dairy sector run mostly by entrepreneurial women and powered by the demand for camel milk from the Somali community. Nevertheless, here too, camel grazing areas and migratory corridors are rapidly being alienated which is a cause of grave concern for camel pastoralists. Unless this issue is addressed by providing secure access to pastures, the sector may be severely affected with dire consequences of rural livelihoods and GDPs of the concerned countries (Köhler-Rollefson and Wanyama, 2022).

Australia is home to a large feral camel population that goes back to the 19th century when camels were brought into the country to help in the colonization of its interior and subsequently released by their Afghan handlers. Australia’s arid environment is ideal for camels and numbers are increasing despite periodical culling initiatives in which thousands of camels are shot by helicopters. From a genetic perspective, the Australian population is a homogenized mixture of different breeds.

While the pastoralists that are the creators of camel biodiversity are losing ground in the remote parts of Africa and Asia, a different process is playing out in the rich countries of the Arabian Peninsula where camel husbandry is becoming industrialized. Large camel dairy farms with thousands of animals are operating in the Gulf countries. Many of the camels originate in Pakistan and belong to the Brela breed that has astonishing milk yields. This breed has been developed by a particular group of Baluch herders in which women play a major role in camel management.

Racing camels and camels for beauty pageants are other important facets of the regional camel industry. Because of the fame and profits associated with these activities, embryo transfer and even cloning individuals with winning traits is becoming routine which is clearly leading to an extreme narrowing of the gene pool (Olsson et al., 2021).

## Conclusions

The strengths of the camel in a planetary scenario of rising temperatures are its drought and heat resilience, ability to convert thorny plants into milk, and general capacity for food production in challenging environments. When camels are kept in industrial systems and fed optimized rations, they lose these qualities. In order to maintain camel genetic diversity, we need to support the pastoralist communities that have developed this asset by providing them with secure grazing rights and access to value chains. This approach would serve the dual goals of in-situ conservation as well as enhancing food security and rural livelihoods. In an ideal world, the wealthy actors whose breeding practices undermine camel biodiversity would extend their support and throw their weight behind the poor communities that conserve it and have created an important part of our human biocultural heritage.

*Conflict of interest statement.* The author is involved in camel conservation in India.

## About the Author

Dr. med. vet. habil Ilse Ulrike Köhler-Rollefson is a veterinarian by training and the coordinator of the League for Pastoral Peoples and Endogenous Livestock Development (LPP) as well as the co-founder of India’s first dedicated camel dairy. She has consulted for diverse clients, such as World Bank, GTZ/GIZ, Misereor, FAO, ILRI, UNDP, and many others. Her work for camel conservation has been awarded by the presidents of both India and Germany, and she is the author of a popular book entitled *Camel Karma: Twenty Years Among India’s Camel Nomads*.
